# ORAL HAIRY POLYP IN A TEENAGER: CASE REPORT AND LITERATURE REVIEW

**DOI:** 10.1590/1984-0462/2020/38/2020143

**Published:** 2020-12-18

**Authors:** Vinícius Gonçalves de Souza, Damilys Joelly Souza Santos, Thalía Rissa Silva, Mathias Rezende Macedo, Tatiana Santos Araújo, Aparecida de Lourdes Carvalho, Ludimila Paula Vaz Cardoso, Carla Silva Siqueira Miranda

**Affiliations:** a Universidade Federal de Jataí, Jataí, GO, Brazil.; b Secretaria Municipal de Saúde de Jataí, Jataí, GO, Brazil.

**Keywords:** Dermoid cyst, Oral medicine, Pathology, oral, Pediatrics, Cisto dermoide, Medicina bucal, Patologia bucal, Pediatria

## Abstract

**Objective:**

To report an unusual case of oral hairy polyp (HP) and review the literature, providing epidemiological, clinical and histopathological information on this disease.

**Case description:**

A 12-year-old male patient was referred to a Stomatology department with a nodule in the posterior midline of the tongue. The patient did not know exactly when it arose or whether it had grown since then. Clinical exam revealed a bulky and mobile pedunculated mass lesion on the dorsum of the tongue, with a diameter of approximately 1 cm. The patient’s mother reported no previous health problem. An excisional biopsy was performed, the surgical specimen was sent for anatomopathological analysis, and the findings were compatible with the diagnosis of HP.

**Comments:**

Hairy polyp is a rare lesion, especially in the oral region. The literature search revealed only 10 case reports of oral HP published between January 1999 and January 2019, and they revealed a predominance of the disease in female newborns. Two uncommon facts were presented in this case: the patient was male and diagnosis was made at 12 years old.

## RESUMO

**Objetivo:**

Relatar um caso incomum de pólipo piloso (PP) oral e revisar a literatura para trazer informações epidemiológicas, clínicas e histopatológicas acerca da doença.

**Descrição do caso:**

Paciente do sexo masculino, 12 anos de idade, encaminhado ao Departamento de Estomatologia com nódulo na região posterior de linha média da língua. O paciente não soube relatar quando surgiu a lesão e se ela havia crescido desde então. O exame clínico revelou massa pedunculada, volumosa e móvel no dorso da língua, medindo aproximadamente 1,0 cm de diâmetro. A mãe do paciente relatou que ele nunca havia tido nenhum problema de saúde anterior. Foi realizada biópsia excisional e o material foi enviado para análise anatomopatológica, sendo os achados compatíveis com o diagnóstico de PP.

**Comentários:**

O pólipo piloso é uma lesão rara, especialmente na região oral. A pesquisa bibliográfica revelou apenas dez relatos de casos de PP oral, publicados entre janeiro de 1999 e janeiro de 2019, sendo observada predominância da doença em recém-nascidos do sexo feminino. Dois fatos incomuns ocorreram neste caso: tratava-se de um paciente do sexo masculino e o diagnóstico se deu aos 12 anos de idade.

## INTRODUCTION

Congenital tumors of the oral cavity are rare manifestations, often diagnosed in the first years of life. One of these is hairy polyp (HP), a benign lesion composed of pedunculated masses of mesodermal and ectodermal origin, usually from the nasopharynx or oropharynx, and covered with skin with sebaceous glands and hair.^1^

Clinical manifestations of HP depend on its location and size, but commonly cause respiratory symptoms and feeding difficulties.^2^ HPs may be diagnosed through clinical exams, histopathological analysis of the mass, and imaging exams, including computed tomography and magnetic resonance scan.^3^ ^,^ ^4^ HPs are treated with complete excision of the mass, with no need for complementary treatments.^1^

The present paper is a case report of a 12-year-old male patient with a HP in the oral cavity, whose diagnosis was confirmed by anatomopathological analysis of the surgical specimen. It also presents a literature review of the common characteristics of this lesion.

## CASE DESCRIPTION

The patient reported was escorted to the Stomatology Department, where clinical examination and biopsy were performed. The surgical material was sent to the pathology service where it was entirely and routinely processed for paraffin embedding and staining with hematoxylin and eosin (H&E). The case report was approved by the institutional review board.

The 12-year-old male patient was referred to the Stomatology Department because he noticed a nodule in the posterior midline of the tongue during self-examination, although he did not know exactly when it arose or whether it had grown since then. Clinical exam revealed a bulky and mobile pedunculated mass lesion on the dorsum of the tongue, with a diameter of approximately 1 cm, displaced to the posterior part of the oral cavity, in close contact with the oropharyngeal region, with color and texture similar to the adjacent mucosa. The patient’s mother reported that he had never had any previous health problem.

**Figure 1:**
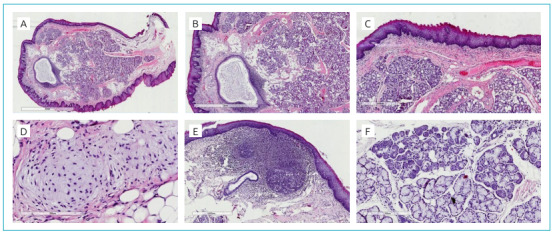
(A) Polypoid lesion with orthokeratinized cyst (4× magnification). (B) Keratinized stratified squamous epithelium with indentations that suggest the formation of skin adnexa and proliferation of a variety of tissues (10× magnification). (C) Proliferation of connective tissue permeating areas of glands and lipids (20× magnification). (D) Hyaline matrix and chondroblasts compatible with cartilage tissue, and permeating adipocytes (40× magnification). (E) Active lymphoid tissue. (F) Serous and mucous salivary glands with normal aspect (20× magnification).

The lesion was excised under local anesthesia without complications. The surgical specimen was sent for anatomopathological analysis supervised by an oral pathologist. Microscopic analysis revealed a polypoid lesion covered with keratinized stratified squamous epithelium, containing indentations along its entire length, which suggested the formation of skin adnexa and the presence of a variety of tissues, including seromucinous salivary glands, cartilage tissue, lymphoid hyperplasia, muscle and adipose tissue. All tissues were mature and had no degree of atypia (Figure 1). The histopathological findings were compatible with diagnosis of HP. The postoperative period was without complications and the patient was followed for 18 months and showed no clinical signs of tumor recurrence. The case report was approved by the institutional review board under number #2.283.697 in September 19^th^, 2017 (Federal University of Goiás).

## DISCUSSION

A literature review was carried out in order to survey case reports published between January 1999 and January 2019 that focused only on the oral region. Thus, articles that presented case reports of HPs in the nasopharynx and oropharynx regions were not considered. Only cases of bigerminal lesions (of ectodermal and mesodermal origin) that were similar to the classic description of HP/dermoid cyst presented in Arnold’s classification^5^ were included (Table 1).

**Table 1:** Arnold’s taxonomy of germ lesions*.

Classification	Description
Dermoids (including hairy polyps)	Derived from epidermal and mesodermal germ layers. A fatty tissue is predominant in the matrix.
Teratoids	A poorly differentiated tissue derived from the three germ layers.
Teratomas	More differentiated tissue with trigerminal origin. Organoid structures can be identified histologically.
Epignathic	A parasitic fetus with trigerminal layer and usually incompatible with life

*Adapted from Ibrahim et al.^5^

The search was performed in databases PubMed, Centro Latino-Americano e do Caribe de Informação em Ciências da Saúde (BIREME) and Scientific Electronic Library Online (SciELO) by pairing the descriptors “hairy polyp” and “dermoid cyst” with “mouth” and “oral”, using the “and” connector.

From the total of 1,661 articles found in the initial search, two different authors independently selected the original publications in English that presented case reports of human HP in the oral cavity, excluding duplicates. In addition, the bibliographic references were checked in order that any reports not found in the initial search which fit the inclusion criteria could be added. Disagreements between evaluators were resolved by a third investigator during the selection process.

**Figure 2:**
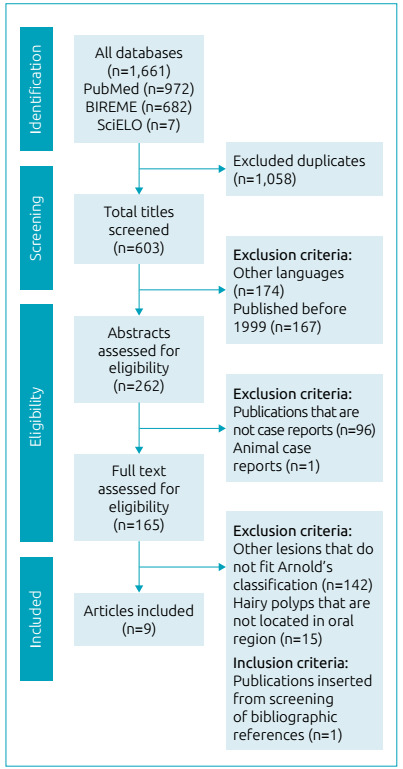
Flow chart of literature search.

A total of nine articles were included in this review. The selection process and search results are described in the flowchart in Figure 2. For case series, only those cases that met the inclusion criteria were included. After reading of the full-text articles and identification of the cases presented, 10 cases of HP in the oral region were chosen for analysis in the present study.

Two authors independently extracted and assessed the following information: surname of the first author, gender and age of the patient, location of the lesion, symptoms, histopathological and other findings and associated complications. When incompatible information was extracted by the two authors, a third author carried out the analysis to ensure data integrity.

Cases of HP are rare, and lesions are usually located in the nasopharynx or soft palate and identified in the first years of life.^4^ ^,^ ^5^ ^,^ ^6^ ^,^ ^7^ The incidence of HP is six times higher in women.^3^ ^,^ ^8^ ^,^ ^9^ Four out of 10 cases of oral HP reported in the present review occurred in men (Table 2).^10^ ^,^ ^11^ ^,^ ^12^ Although the lesions are often early diagnosed, some cases are late diagnosed due to lack of symptoms,^12^ as that reported herein.

**Table 2:** Epidemiology and clinical findings of hairy polyps of the oral cavity reported in the last 20 years (from January 1999 to August 2019).

Citation	Gender/age	Location origin	Symptom
Tariq et al.^12^	Male/12 years	Soft and hard palate	Not reported
Tariq et al.^12^	Male/1 month	Lower lip	Not reported
Desai et al.^2^	Female/6 months	Hard palate midline, prevalent in the left side	Asymptomatic
Puricelli et al.^4^	Female/3 months	Anterior midline dorsum of tongue, at an almost equal distance between the apex of the tongue and the foramen cecum	Difficulty in swallowing and breathing, associated with cough and vomiting
Yilmaz et al.^1^	Female/2 months	Right side of the soft palate	Difficulty in breathing
Herlin et al.^11^	Male/47 years	Deep surface of the upper lip, bilateral	Asymptomatic
Gokul et al.^13^	Female/8 months	Hard palate, prevalent in the left side	Nasal regurgitation, difficulty in feeding
Erdogan et al.^14^	Female/40 days	Tongue lateral	Not reported
Kiroglu et al.^15^	Female/1 day	Midline of hard palate	Difficulty in feeding
Mahmood et al.^10^	Male/35 years	Midline dorsum of tongue	Discharge of grayish-yellow material

The present study reports an unusual case of oral HP in a 12-year-old male patient, located on the posterior dorsum of the tongue, one of the rarest sites of occurrence of this type of lesion.^4^ The symptoms of HP depend on its location and extent, but as it is a congenital malformation associated with obstruction of upper airways, it can cause respiratory distress, choking episodes, cyanosis, and feeding difficulties.^6^ ^,^ ^7^ HPs can also be asymptomatic, and are most often completely resolved after surgery.^12^ ^,^ ^13^ The present review describes three cases of HP in the tongue,^4^ ^,^ ^10^ ^,^ ^14^ two asymptomatic cases,^2^ ^,^ ^11^ and three cases with no reports of the presence or absence of symptoms.^10^ ^,^ ^12^ ^,^ ^14^

It is worth noting that most lesions are single and unilateral,^3^ ^,^ ^9^ and they occur 6.5 times more frequently on the left side.^1^ ^,^ ^6^ ^,^ ^7^ The lesion described here was single, pedunculated and located in the midline of the tongue,^15^ which made it simple for the mass to be excised without the need for preoperative imaging.^1^ ^,^ ^3^ There are some reported cases of multiple lesions in newborns and adults, but these are considered rare.^9^ The present review reports only one case of bilateral HP.^11^

Although HPs are considered as rare lesions, especially in the oral cavity, they are the most common congenital benign tumors of the naso-oropharyngeal region.^6^ ^,^ ^7^ ^,^ ^12^ The lesion cannot be detected at early stages due to its size, location, and the lack of symptoms, suggesting that it can be subdiagnosed or diagnosed by chance during routine exams.^16^ The lesion reported here was isolated, not associated with congenital alteration or genetic predisposal, as in most cases reported in the literature.^3^ ^,^ ^6^ ^,^ ^12^ However, studies have associated HP with other congenital anomalies, such as tongue bifurcation, cleft palate, agenesis of the external ear, ankyloglossia, among others.^1^ ^,^ ^12^ ^,^ ^16^

No theory has explained the exact origin of HP, but some authors suggest that it is related to malformations of the first and second branchial arches, Eustachian tube and/or middle ear.^7^ ^,^ ^17^ During embryonic development, the endodermis of the first branchial arch expands into the middle ear with mesenchymal cells of the neural crest. Then, the ear tube stimulates the mesenchymal cells to transform into the epithelium that coats the upper half of middle ear, while the mesoderm expands into the middle ear to coat its lower half. These cells can transform into a HP if their differentiation is interrupted, and they then settle in the middle ear, Eustachian tube, or along any part of the branchial archs.^7^

The classification of HPs varies due to the difficulties to define the lesions. The differential diagnosis of HP includes benign teratoma, dermoid cyst, and choristoma.^6^ ^,^ ^17^ HPs are exclusively composed of elements from the ectodermal and mesodermal embryonic leaflets^6^ ^,^ ^7^ ^,^ ^18^ and differ from teratoma in that the latter presents trigeminal lesions (ectoderm, mesoderm, and endoderm) with varied degrees of differentiation.^18^ Both HPs and dermoid cysts have ectodermal and mesodermal germ layers, with the presence of cysts and keratinized epithelium; however, dermoid cysts predominantly have the mesoderm layer.^19^ This is the closest classification criterion based on morphology and origin. A choristoma is generally composed of one normal and mature tissue located in an anatomically different region.^1^ ^,^ ^18^

The histopathological analysis revealed mesodermal and ectodermal histologic structures that are usually found in HP lesions, corroborating literature reports:^6^ ^,^ ^7^ ^,^ ^18^ Lymphoid hyperplasia and a variety of mature tissues such as cartilage, muscle and adipose tissues, orthokeratinized cyst, seromucinous salivary glands, and indentations suggested the formation of skin adnexa (Table 3).

**Table 3:** Histopathologic findings of hairy polyps of the oral cavity reported in the last 20 years (from January 1999 to August 2019).

Citation	Histopathologic findings
Tariq et al^12^	Covered with keratinized pseudostratified squamous epithelium; skin adnexa and seromucinous glands
Tariq et al.^12^	Covered with keratinized pseudostratified squamous epithelium; skin adnexa, adipose tissue, cartilage and mature bone
Desai et al.^2^	Immature hair follicles; sebaceous and sweat glands in the fibrous connective tissue
Puricelli et al.^4^	Keratinized squamous epithelium containing connective and adipose tissue, smooth and striated muscle, and salivary glands
Yilmaz et al.^1^	Fibrous and adipose tissue, seromucinous glands, hair follicles, eccrine sebaceous and sweat glands, keratinized stratified squamous epithelium
Herlin et al.^11^	Aspect compatible with dermoid cyst (dermal and epidermal tissues)
Gokul et al.^13^	Parakeratinized squamous epithelium, hair follicles, sebaceous and sweat glands in fibrous stroma; adipose tissue
Erdogan et al.^14^	Stratified squamous epithelium, adipose tissue, muscle, skin adnexa, minor salivary glands, and cartilage
Kiroglu et al.^15^	Keratinized squamous epithelium, pilosebaceous glands, adipose and connective tissue
Mahmood et al.^10^	Stratified squamous epithelium, sebaceous glands, apocrine glands, and cartilage.

HPs have no malignant potential and are treated by total excision of the lesion.^1^ ^,^ ^17^ ^,^ ^20^ There is low probability of tumor recurrence after its surgical removal,^6^ ^,^ ^20^ except for a few reported cases in which tumor location hampered its complete excision.^20^ In this sense, the postoperative follow-up is essential to check the healing process and early detect a possible recurrence, as well as to examine the functional response of the tongue in some patients who are in the developmental period^4^. Our patient did not present clinical signs of tumor recurrence after one and a half year of follow-up.

Knowledge on HPs is relevant due to the fact that they are rare lesions, especially in the oral region. It is vital to exclude other diagnostic hypotheses, as well as to prevent respiratory distress and other complications.
